# Breaking social media fads and uncovering the safety and efficacy of mouth taping in patients with mouth breathing, sleep disordered breathing, or obstructive sleep apnea: A systematic review

**DOI:** 10.1371/journal.pone.0323643

**Published:** 2025-05-21

**Authors:** Jess Rhee, Alla Iansavitchene, Sonya Mannala, M. Elise Graham, Brian Rotenberg

**Affiliations:** 1 Department of Otolaryngology – Head and Neck Surgery, London Health Sciences Centre, London, Ontario, Canada; 2 Department of Library Services, London Health Sciences Centre, London, Ontario, Canada; 3 University of Saskatchewan College of Medicine, Saskatoon, Saskatchewan, Canada; Xuzhou Central Hospital, The Xuzhou School of Clinical Medicine of Nanjing Medical University, CHINA

## Abstract

**Background:**

Social media has contributed to a potentially unsafe trend of nighttime mouth taping for individuals with mouth breathing, sleep disordered breathing, or sleep apnea as a home remedy to treat these issues. This systematic review is aimed to highlight any potential benefits or harms with this practice.

**Methods:**

A comprehensive librarian-designed literature search was performed using PRISMA guidelines. Using search terms, “mouth taping, adhesive mouthpiece, porous oral patch, surgical tape, breathing mouthpiece, sleep, microsleep, breath, breathing, or mouth breathing”, MEDLINE, Embase, and Google Scholar were searched from February 1999 to February 2024. Covidence software was used for screening and data entry performed into a data collection sheet designed *a priori.*

**Results:**

Covidence software was utilized to screen 120 articles. After 34 duplicates were removed, 86 articles were screened by two independent reviewers. Sixty-two were excluded. Twenty-four went on to full text review and 10 met inclusion criteria with a total of 213 patients. Two studies showed statistically significant improvement in established markers of sleep apnea such as apnea-hypopnea index (AHI) or oxygen desaturations. Other studies showed that mouth taping offered no differences and even discussed potential risks including asphyxiation in the presence of nasal obstruction. Many studies excluded anyone with nasal obstruction or pathology.

**Conclusion:**

The social media trend of mouth taping for individuals with mouth breathing, sleep disordered breathing, or sleep apnea has been reviewed. Based on the data presented by these 10 different studies, it seems that there is a potentially serious risk of harm for individuals indiscriminately practicing this trend. Further studies are required to elucidate any clinical benefit this practice may have.

## Introduction

Obstructive sleep apnea (OSA) is characterized by interruptions of breathing during sleep and is considered the extreme end of sleep disordered breathing (SDB) [1]. OSA causes oxygen desaturation events and, depending on the severity, can cause long-term sequelae including hypertension and cardiovascular, pulmonary, and quality of life detriments [1,2]. Mouth breathing has been identified as a risk factor for OSA [1,2]. Mouth breathing also worsens OSA by narrowing the airway and increasing obstruction [1,2]. Sleep disordered breathing (SDB) or OSA in children is typically managed through adenotonsillectomy [3]. In adults, OSA is usually treated with a continuous positive airway pressure (CPAP) machine [2,4]. However, for many individuals, CPAP adherence is poor due to discomfort [4].

Mouth breathing occurs when either nasal or pharyngeal obstruction compels individuals to switch from nasal breathing to breathing through the mouth. Allergic rhinitis, adenoidal and tonsillar hypertrophy and deviation of the nasal septum are among the most common causes for mouth breathing [5]. Nasal obstruction and the resultant mouth breathing has also been implicated in SDB and OSA [6,7], with SDB considered to be both a cause and a consequence of nasal obstruction.

Many interventions have been considered to address mouth breathing. One such intervention that has increased in popularity likely due to social media trends is the phenomenon of mouth taping. This involves participants maintaining mouth closure by occlusion with tape while sleeping to prevent mouth breathing. Participants allege benefits ranging from better sleep quality to anti-aging properties to improvements in dry mouth, bad breath, and concentration [8], but lack of concrete evidence gives rise to concern about this practice both from a safety and effectiveness perspective.

The aim of this study was to investigate the literature to determine the effects of mouth taping on SDB and OSA to assess if this practice carries meaningful benefit and/or risk of harm.

## Methods

A comprehensive search strategy was devised with an assistance of a clinical librarian (AI) with experience in conducting searches in electronic databases. Adhering to Preferred Reporting Items for Systematic Reviews and Meta-Analyses (PRISMA) guidelines, the systematic search strategy was tailored to our predefined inclusion and exclusion criteria and conducted using MEDLINE® and Embase® (both via the OVID platform) electronic databases. The web-based search engine Google Scholar was searched to identify additional potentially relevant non-indexed articles in bibliographic databases. References of all studies identified as applicable for inclusion were reviewed for additional articles relevant to our systematic review.

Systematic literature searches were carried out from February 1999 until February 2024. To identify relevant studies, we utilized a sensitive search strategy comprised of the following search terms (using combinations of subject headings (i.e., MeSH in MEDLINE) and keywords): mouth taping, adhesive mouthpiece, porous oral patch, surgical tape, breathing mouthpiece, sleep, microsleep, breath, breathing, or mouth breathing with further filtering to adverse effects. English language restriction was applied. The search strategies were modified using appropriate thesaurus terms and fields suitable for each database.

A detailed description of our search strategy can be found in supplementary appendix (S1 Fig). Identified records from the electronic searches were downloaded and imported into Covidence systematic review software (Veritas Health Innovation, Melbourne, Australia https://www.covidence.org/). Abstract and full text review as well as data extraction were performed in duplicate by two reviewers (S.M., J.R.).

**Fig 1:**
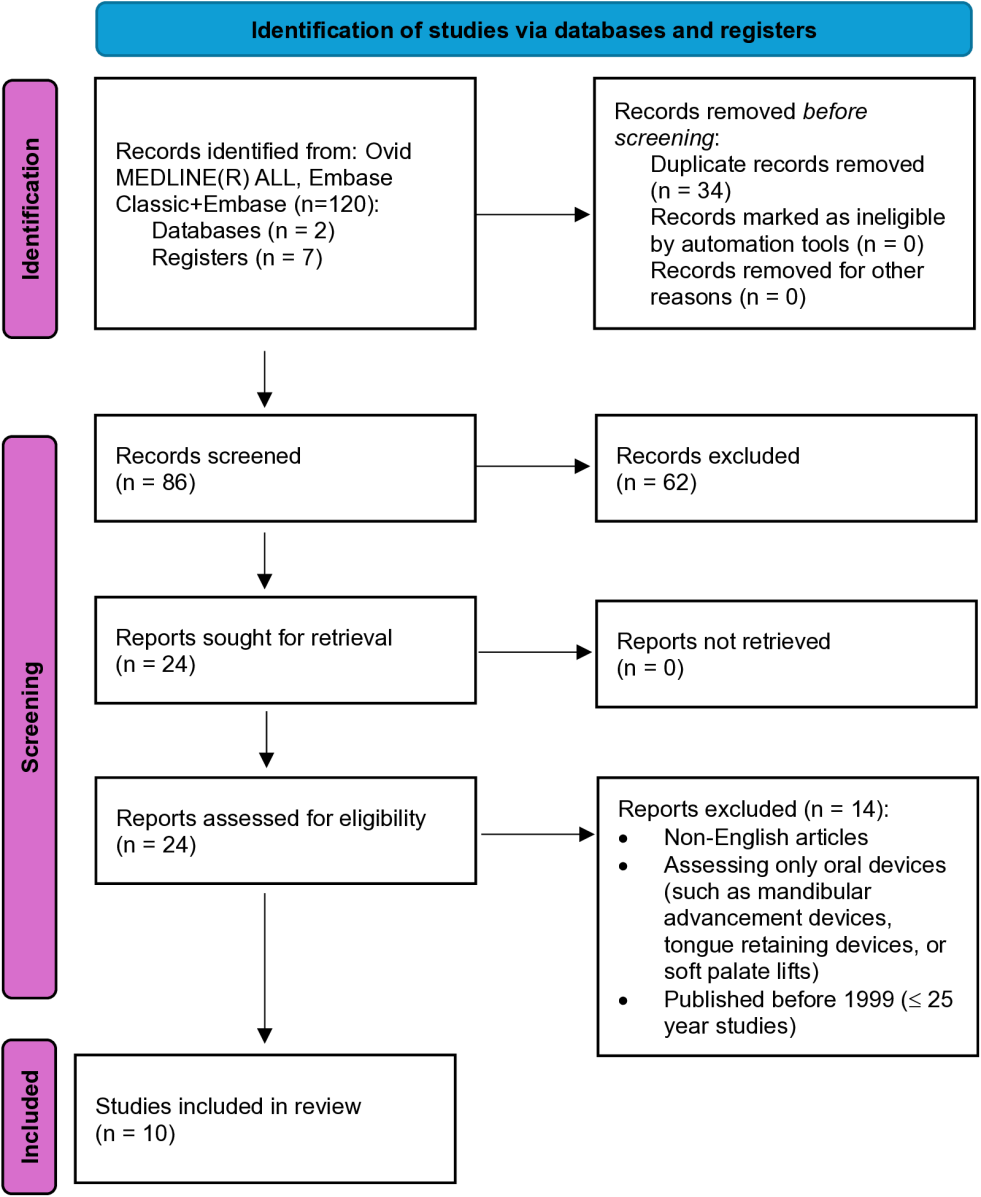
Preferred Reporting Items for Systematic Reviews and Meta-Analyses (PRIMSA) flow diagram for systematic reviews.

We employed the following inclusion criteria: all pediatric and adult patients with OSA, nasal obstruction, or mouth breathing during sleep; and oral/mouth taping, or any similar devices such as oral porous patch and chinstraps. We considered randomized control trials and prospective studies only, with objective and subjective outcomes. The exclusion criteria included non-English articles, only utilizing an oral devices such as mandibular advancement devices without mouth taping, tongue retaining devices, or soft palate lifts. Studies also had to be published within the last 25 years. Initially, 120 studies were identified. After automatic removal of 34 duplicates, 86 abstracts were screened, with 24 studies undergoing full-text screening. A total of 10 studies met inclusion criteria and were included in this systematic review (Fig 1).

Our study protocol was registered with PROSPERO (International Prospective Register of Systematic Reviews) under the following identifier number CRD42024509650.

## Results

### Baseline characteristics

From the included studies, six were prospective cross sectional studies, one was a randomized control trial, one was a prospective cohort study, one was a retrospective cohort study, and the last was a prospective crossover study. Eight out of the 10 utilized either adhesive tape or a sealing device to occlude the mouth. Two of studies utilized a chin strap to hold the mouth closed. Labarca et al. utilized a mouth seal as well as a mandibular advancement device (MAD) [9]. Osman et al. utilized a mouth seal as well as a chin strap and a novel nasal spray [10]. Two studies were completed by the same first author in the same year (Jau et al. 2023).

The mean or median age of each study differed, ranging between 38–64 years of age. Sample sizes for studies ranged from 9 to 71 participants, with 233 total patients across all studies included in this systematic review. The mean or median body mass index (BMI) ranged from 24 to 35. Mean or median baseline apnea-hypopnea indexes (AHI) were 13–47. Inclusion and exclusion criteria varied but four studies (Lee et al., Huang et al, Labarca et al, Osman et al), excluded patients with any form of nasal obstruction [2,4,9,10]. Only two studies (Lee et al, Huang et al) included patients with AHI less than 15. Other baseline characteristics are summarized in Table 1.

**Table 1:** Baseline characteristics of selected studies.

Study	Intervention type	Study Type	Mean/Median Age	Sample Size	Inclusion Criteria	Exclusion Criteria	mean/median BMI	Additional Baseline Characteristics
Lee at al. 2022	Mouth seal	Retrospective Cohort Study	median: 38	20	20-60 years of age	Retrognathia	median: 24.5	
					BMI < 30	Allergy to mouth tape		
					AHI < 15	Intolerance of mouth sealing		
					Sleep disordered breathing symptoms	Comorbidies		
					Witnessed mouth breathing during sleep	Tonsils grade 3/4		
					Dry mouth in the morning	Previous nose, palate, or tongue surgery		
						Shift workers		
						(Those with nasal obstruction received nasal sprays)		
Madeiro et al. 2019	Mouth seal	Prospective Cross Sectional Study	mean: 63	13	18-80 years of age	BMI > 40	mean: 30.4	Mean neck cirumference: 41 cm
					patients adapted to oronasal CPAP usage (>3 mo and > 4h/d usage)	Home O2 usage		Mean ESS: 12
								Mean AHI: 43
								Mean duration of CPAP usage: 5 years
								Mean CPAP level: 10.5 cmH2O
Bachour et al. 2003	Chin strap	Prospective Cohort Study	mean: 53.7	15	Observed mouth leak		mean: 31	Neck circumference: 42.6 cm
					Dry mouth in the morning			Mean CPAP level: 9.4 cmH2O
					Nasal obstruction with CPAP			
Huang et al. 2015	Mouth seal	Prospective Cross Sectional Study	mean: 46	30	Patients with snoring and mouth breathing during sleep	Palate position grade 3/4	mean: 26.8	Tonsil grade1/2: 19/11
					AHI 5–15	Tonsils grade 3/4		Uvula grade 1/2: 3/27
						Uvula grade >2		Palate grade 1/2: 12/18
						Allergic rhinitis		
						Chronic rhinitis		
						Septal devtiation		
						Sinonasal disease		
						Facial hair		
						BMI > 30		
Teschler et al. 1999	Mouth seal	Prospective Cross Sectional Study	mean: 64	9	Discomfort due to nasal leak on home nasal bilvel ventilatory support		mean: 24	Mean AHI: 13
					Significant mouth leak during ventilatory assitance in the lab			Mean FEV1: 33% predicted
								Mean FEV1/VC: 52%
								Mean PaCO2/PaO2: 61/57
								IPAP/EPAP level: 17/6 cmH2O
Jau et al. 2023	Mouth seal	Prospective Cross Sectional Study	mean: 45.01	71		Psychiatric disease	mean: 26.8	Mean neck circumference: 39.25 cm
						Neurological disorders		Mean PSQI: 8.63
						Diabetes		Mean ESS: 10.75
						Chronic renal diseases		
						Cancer		
						Cardiovascular diseases		
						Cigarette or alcohol addition		
						Sleep disorders		
Bhat et al. 2015	Chin strap	Prospective Cross Sectional Study	median: 48	26	AHI >=5		median: 31	median mallampati score: 4
								median neck circumference: 16.5in
								Median percentage of total sleep time with SpO2 below 90%: 2.1
								CPAP/bilevel pressure level: 10/10 cmH2O
Labarca et al. 2022	Mouth seal (+ mandibular advancement device)	Prospective Crossover Study	mean: 60.1	21	21-70 years of age	Current benzodiazepine, hypnotic, or opioid usage	mean: 26.81	mean neck circumference: 17.47in
					BMI < 38	Other sleep disorders - insomnia, nacrolepsy, central sleep apnea, parasomnia		mean AHI: 24.35
					Neck circumference <20in for men, < 17in for women	Failure to breathe comfortably through the nose		
					AHI 10–50	Allergy to adhesives		
					Patients with confirmed OSA and usage of a mandibular advancement device of any kind			
Osman et al. 2024	mouth seal + chin strap (+ novel nasal spray)	Randomized Control Trial	mean: 59	10	>=18 years of age	Impaired breathing - nasal congestion/obstruction	mean: 35	mean AHI: 47
								mean neck circumference: 41 cm
								mean ESS score: 7
								mean Insomnia Severity index: 9
Jau et al. 2023	mouth seal	Prospective Cross Sectional Study	mean: 43	18	23-57 years of age	Chronic diseases - psychiatric, neurological, diabetes, chronic renal diseases, cancers, and cardiovascular	mean: 26.96	mean neck circumference: 38.55 cm
					OSA-associated symptoms - snoring and daytime sleepiness	Cigarette or alcohol addiction		mean Pittsburgh sleep quality index: 7.56
						Known sleep disorders		mean ESS score: 9.67

### Primary outcomes

Primary outcomes are summarized in Table 2. Six studies assessed AHI before and after implementation of their form of oral occlusion. Only two of these studies (Lee et al. and Huang et al.) reported a significant decrease in AHI post-occlusion [2,4]. Lee et al. reported a significant reduction in median AHI from 8.3 to 4.7 per hour after tape, and Huang et al. reported a statistically significant reduction in median AHI from 12 to 7.8 per hour after oral patch [2,4]. Three (Bhat et al., Labarca et al., and Osman et al.) did not detect a significant change in AHI [9–11]. Labarca et al. also compared the AHI in patients with MAD plus mouth taping to those utilizing just MAD alone and did report a statistically significant decrease in the median AHI with MAD alone compared to combined treatment from 10.5 to 5.6 per hour [9]. Osman et al. showed no significant difference between patient on a placebo spray compared to mouth taping. There was also no significant difference between the novel nasal spray plus mouth taping to spray alone [10]. The last study looking at AHI was one of the two studies by Jau et al. This group assessed and validated a “mouth puffing” device while patients’ mouths were taped. Jau et al. define mouth puffing as a derivation of mouth breathing while patients’ mouths are occluded with tape [1,12]. As such, they utilized accelerometers on the sides of both cheeks to detect the mouth puffing phenomenon while patients’ mouths were occluded with tape [1]. They found that AHI was significantly reduced in individuals who had no mouth puffing compared to both side or complete mouth puffing [1]. This study reported that AHI was highest in intermittent mouth puffing compared to both non and complete mouth puffing patients [1].

**Table 2:** Primary outcomes for selected studies.

Study	AHI	Snoring Index (SI)	O2 Desat Index (ODI)	Mean Saturation	CPAP pressure	Airlow and Upper Airway Dimensions after flow route change (oronasal to nasal and vice-versa)	Airflow and Upper Airway Resistance	Potential Complications
Lee at al. 2022	Median AHI: 8.3 before tape vs 4.7/hr afer tape (p = 0.0002)* Supine AHI: 9.4 before tape vs 5.5/hr after tape (p = 0.0001)* Non-supine AHI: 3.2 before tape vs 0.6/hr after tape (p = 0.03)*	Median SI: 303.8 before tape vs 121.1 after tape (p = 0.0002)*	Median ODI: 8.7 events/hr before tape vs 5.8 events/hr after tape (p = 0.0003)*	Median: 95 before tape vs 95 after tape				“Mouth-taping is not recommended for moderate or severe OSA patients because it may impose dangers rather than benefits in these patients.”
Madeiro et al. 2019					13 cm H2O for oronasal CPAP pressure vs 12 cm H2O for nasal CPAP pressure (p = 0.039)*	Retropalatal area from nasal to oronasal CPAP: 1109–624 pixels (p = 0.001)* Retroglossal area from nasal to oronasal CPAP: 6677–4934 pixels (p = 0.005)* Retropalatal area from oronasal to nasal CPAP: 723–1198 pixels (p = 0.028)* Retroglossal area from oronasal to nasal CPAP: 4876–5617 pixels (p = 0.012)*	Nasal to Oronasal Flow: Mean peak flow (L/s) significantly decreased from nasal to oronasal route (p = 0.011)* After taping, mean peak flow significantly increased (p = 0.015)*	
Bachour et al. 2003		Mean snoring: 6.7 to 24% of total sleep time (TST) after chinstrap (p < 0.005)*	Mean ODI at 4%: 13 to 5.7 after chinstrap (no sig change)					“As with any mouth occlusion, the possibility exists of aspiration of stomach contents should the patient regurgitate during the night and be unable to expel the emesis.”
Huang et al. 2015	Median AHI: 12 to 7.8/hr after oral patch (p < 0.01)*	Median SI: 146.7 to 40/hr after oral patch (p < 0.01)						“Although patients, on average, improve with treatment, the study does not define the safety or efficacy due to a small single-institution case series without a control group. Further large studies with multi-institution and control groups can be conducted to assess the efficacy and safety of the POP device.”
Teschler et al. 1999								“The authors do not at this stage advocate taping the mouth for indiscriminate long-term home use, because of the risk of asphyxia in the presence of nasal obstruction, machine or power failure, or regurgitation.”
Jau et al. 2023			Mean ODI: 16.3 to 30.5 events/hr after tape (p = 0.037)*					
Bhat et al. 2015	Median AHI: no significant difference after chinstrap (although patients on optimal CPAP settings compared to diagnostic PSG settings had significant improvement of AHI)							
Labarca et al. 2022	Median AHI in MAD + tape compared to MAD alone: 5.6 vs 10.5/hr (p = 0.02)* No significant difference comparing tape to baseline alone							
Osman et al. 2024	No significant difference comparing placebo vs spray + tape No significant difference comparing spray + tape vs spray alone							
Jau et al. 2023	Mean AHI: AHI/min was significant reduced during non-mouth puffing (NMP) compared to side mouth puffing (SMP) or complete mouth puffing (CMP) (p < 0.001 for both)* Intermittent mouth puffing (IMP) mean AHI: AHI/min was significant higher in IMP compared to both NMP and CMP (p < 0.001 for both)*		Mean ODI: ODI/min was significantly lower in CMP compared to SMP (p < 0.001)* Significantly lower in NMP compared to SMP and CMP (p < 0.001 for both)* Significantly higher in IMP compared to SMP, CMP, and NMP (p < 0.001 for all)*					
**Study**	**Mouth leak**	**Arousal Index**	**Cephalometry**	**Transcutaneous CO2 Tension (Ptc,CO2)**	**Sleep state/REM sleep/Total sleep time**	**Correlation Studies**	**Percentage of SpO2 under 90% (T90)**	
Lee at al. 2022								
Madeiro et al. 2019								
Bachour et al. 2003	Mean mouth leak: 42.9% to 23.8% total sleep time (TST) after chinstrap (p < 0.05)*	Mean arousal index: 33.4 to 23.6/sleep hour after chin strap (p < 0.05)*	Ant lower facial height: 79.3 to 70.4mm after chinstrap(p < 0.05)* Nasion and menton distance: 133.8 to 125mm after chinstrap (p < 0.05)* Ant cranial baseline and mandibular line angle: 36 to 32.8 degrees after chinstrap (p < 0.05)* Base of tongue and post pharyngeal wall distance: 7.8 to 9.7mm after chinstrap (p < 0.05)* Tip of uvula and post pharyngeal wall distance: 2.3 to 6.4mm after chinstrap (p < 0.05)*					
Huang et al. 2015			Retropalatal space: 7.4 to 8.6mm after oral patch (p < 0.05)* Retrolingual space: 6.8 to 10.2mm after oral patch (p < 0.05)*					
Teschler et al. 1999	Median: 0.35 to 0.06 L/s after tape (p = 0.05)*	No sig change		No sig change	Rem sleep %: 12.9 to 21.1% after tape (p = 0.0016)*			
Jau et al. 2023	Mean mouth puffing: Mean intermittent mouth puffing changed from 19.06 to 26.47% (p = 0.037)*					Intermittent mouth puffing was positively associated with uvula length: 0.301 (p < 0.05)* ODI was negatively associated with the min width of the airway and nasal width: -0.357 and -0.381, respectively (p < 0.05)* T90 was negatively associated with the min width of the airway and nasal width: -0.474 and -0.316, respectively (p < 0.001)*		
Bhat et al. 2015					REM sleep % comparing diagnostic PSG to chinstrap: 20.2 to 8.7% (p = 0.0025)* REM sleep % comparing optimal CPAP to chinstrap: 25.5 to 8.7% (p = 0.0025)* Total sleep time (TST) comparing diagnostic PSG to chinstrap: 270 to 136.3mins (p < 0.001) TST comparing optimal CPAP to chinstrap: no signifcant change		No significant difference in SpO2 nadir after chinstrap	
Labarca et al. 2022								
Osman et al. 2024		Mean Arousal Threshold comparing spray + tape vs placebo: 142 vs 130/hr (p < 0.05)*						
Jau et al. 2023	Mild-Mod OSA: Significantly more NMP and IMP % compared to normal individuals (p < 0.001 for both)* Severe OSA: Significantly more NMP and IMP% compared to mild-mod OSA and normal individuals (p < 0.001 for all)*							

Snoring index (SI) was assessed by three studies. Lee at al., Bachour et al., and Huang et al. all reported a significant decrease in SI after mouth taping or chinstrap [2,4,13].

Oxygen desaturation index (ODI) was assessed by four studies. Two studies (Lee at al. and Jau et al.), reported a statistically significant decrease in ODI after mouth taping [4,12]. Bachour et al. did not find a significant decrease in ODI with chinstrap usage [13]. The second study by Jau et al. found a significantly lower ODI in those with their mouths taped with complete mouth puffing compared to side mouth puffing [1]. This study also reported significantly lower ODI in individuals with no mouth puffing compared to both side and complete mouth puffing. Lastly, the study reported that ODI was significantly higher in intermittent mouth puffing compared to side, complete, and non-mouth puffing patients [1].

Mean oxygen saturation was only assessed in one study (Lee at el.) with no significant difference with mouth taping [4].

Mean continuous positive airway pressure (CPAP) levels were assessed by Madeiro et al. and showed a significant reduction in pressure levels (cm H_2_O) when comparing the oronasal and nasal CPAP plus mouth tape pressures [14].

Mouth leak was assessed by four studies. Mouth leak is defined as the air pressure that is lost from nasal CPAP because of patients opening their mouths during sleep which can cause upwards of 10–15% of pressure lost as leakage [13,15]. Bachour et al., Huang et al., and Jau et al. all reported that mouth leak was significantly reduced as a percentage of time slept or volume flow rate (L/s) after oral occlusion [2,12,13]. The second study by Jau et al. grouped mouth taped patients into mild to moderate OSA or severe OSA groups, and reported significantly more non and intermittent mouth puffing in both groups compared to normal individuals [1]. Those with severe OSA also had more non and intermittent mouth puffing compared to mild to moderate OSA patients [1]. This study by Jau et al., also reported significant correlations – a positive correlation between intermittent mouth puffing and uvula length, a negative correlation between ODI and the minimal width of the airway and nasal width, and a negative correlation between the percentage of oxygen saturation (SpO2) under 90% (T90) with the minimal width of the airway and nasal width [1].

Arousal threshold, as defined by the maximum estimated ventilatory drive just before coirtical arousal during NREM (non-rapid eye movement sleep) respiratory events that resulted in arousal, was assessed by Osman et al. Patients in the novel upper airway muscle dilator spray plus tape group compared to placebo had a significantly increased arousal index with reduced events per hour [10]. The study does comment that the spray is an effective upper airway dilator, but the effects may be impaired when the mouth is taped, due to an increased sleep drive secondary to OSA [10].

Transcutaneous carbon dioxide (CO_2_) tension (Ptc,CO_2_), was assessed by Teschler et al., and there was no significant difference with mouth taping [16].

Two studies assessed rapid eye movement (REM) sleep states. Teschler et al. showed that REM sleep percentage significantly increased after mouth tape [16], whereas Bhat et al. showed that REM sleep percentage significantly reduced with chinstrap when compared to diagnostic polysomnography (PSG) study [11]. Although this study only performed chin strapping for the first two hours, the reduction in REM sleep with chinstrap compared to PSG was analyzed as a percentage of the total sleep time [11]. Bhat et al. also demonstrated that REM sleep percentage was significantly lower with patients using chinstraps compared to those on optimal CPAP [11]. Total sleep time was also significantly lower with chinstrap compared to patients during their diagnostic PSG study, although this was an expected result as chin strapping was only used for the first two hours [11]. Total sleep time was unchanged when comparing chinstrap to those on optimal CPAP [11]. Bhat et al. also showed that there was no significant difference in SpO2 nadir after chinstrap usage [11].

Utilizing the Newcastle-Ottawa Scale for assessing the quality and risk of bias of these 10 studies showed that all studies on mouth taping were of poor quality for varying reasons (Table 3) [17].

**Table 3:** Risk of bias assessment (Newcastle-Ottawa Quality assessment Scale Criteria).

	Selection				Comparability	Outcome			Score
Study	**Representativeness of Exposed Cohort**	**Selection of Non-Exposed Cohort from Same Source as Exposed cohort**	**Ascertainment of Exposure**	**Outcome of Interest was Not Present at Start of Study**	**Comparability of Cohorts**	**Assessment of Outcome**	**Follow-up Long Enough for Outcome to Occur (Median Duration of Follow-up ≥ 6 months)**	**Adequacy of Follow-Up**	**Quality Score**
Lee at al. 2022	Participants were a selected group of patients from 2020–2021 at Chang Gung Memorial Hospital, Taiwan with inclusion and exclusion criteria	Yes ★	Medical records/visits ★	Yes ★	What cofounders were adjusted for was not clearly stated	Record linkage – home sleep studies ★	No follow-up	No	Poor
Madeiro et al. 2019	Participants were of a somewhat representative group from 18–80 years of age, well adapted to oronasal CPAP, and those of BMI > 40 excluded ★	Yes ★	Medical visit/formal sleep study ★	Yes ★	What cofounders were adjusted for was not clearly stated	Record linkage – formal sleep studies ★	No follow-up	No	Poor
Bachour et al. 2003	Participants were somewhat representative including patients with observed mouth leak, mouth dryness, and nasal obstruction with CPAP ★	Yes ★	Medical visit/formal sleep study ★	Yes ★	What cofounders were adjusted for was not clearly stated	Record linkage – formal sleep studies ★	Yes ★	Only 6/15 continued with follow-up as suitable candidates, and of those 6, only 4 completed the 6 month follow up	Poor
Huang et al. 2015	Participants were of a selected group with specific inclusion and exclusion criteria	Yes ★	Medical visit/formal sleep study ★	Yes ★	What cofounders were adjusted for was not clearly stated	Record linkage – formal sleep studies ★	No follow-up	No	Poor
Teschler et al. 1999	Participants were somewhat representative with specific inclusion criteria ★	Yes ★	Medical visit/formal sleep study ★	Yes ★	What cofounders were adjusted for was not clearly stated	Record linkage – formal sleep studies ★	No follow-up	No	Poor
Jau et al. 2023	Participants were truly representative ★	Yes ★	Medical visit/home sleep study ★	Yes ★	The study did control for BMI and Angle’s Classification with ANOVA testing ★	Record linkage – home sleep studies ★	No follow-up	No	Poor
Bhat et al. 2015	Participants were truly representative ★	Yes ★	Medical visit/formal sleep study ★	Yes ★	All groups were required to be normally distributed with ANOVA and Student Newman-Keuls testing ★	Record linkage – formal sleep studies ★	No follow-up	No	Poor
Labarca et al. 2022	Participants were of a selected group with specific inclusion and exclusion criteria	Yes ★	Medical visit/formal sleep study ★	Yes ★	The study controlled for baseline characteristic differences using nonparametric statistical tests including Mann-Whitney U and Fisher exact tests ★	Record linkage – formal sleep studies ★	No follow-up	No	Poor
Osman et al. 2024	Participants were truly representative ★	Yes ★	Blinded formal sleep study assessment ★	Yes ★	The study had equal male and female participants ★	Blinded assessment ★	No follow-up	No	Poor
Jau et al. 2023	Participants were of a selected group with specific inclusion and exclusion criteria	Yes ★	Medical visit/formal sleep study ★	Yes ★	What cofounders were adjusted for was not clearly stated	Record linkage – formal sleep studies ★	No follow-up	No	Poor

Table 4 summarizes the secondary/qualitative outcomes assessed by the selected studies. Huang et al. reported that median Epworth sleepiness scale (ESS) and visual analog scale of snoring (VAS) both significantly reduced after mouth sealing [2]. Labarca et al. showed no significant difference in ESS with oral tape and MAD [9].

**Table 4:** Qualitative outcomes for selected studies.

Study	ESS (epworth sleepiness scale)	VAS (visual analog scale of snoring)	Dry Mouth
Huang et al. 2015	Median ESS: 8.1 to 5.2 after oral patch (p < 0.05)	Median VAS: 7.5 to 2.4 after oral patch (p < 0.05)	
Labarca et al. 2022	No significant difference		

## Discussion

Overall, the evidence surrounding the effectiveness of mouth occlusion or chin strapping is uncertain, and conclusions vary in the included studies. PSGs are considered the gold standard for the diagnosis of OSA, and although criticized for its limitations, AHI is a well-established and studied metric of OSA severity [18,19]. Only six of the ten included studies assessed AHI as a primary outcome, and only five of those compared AHI directly between interventions. Only three of these studies showed a statistically significant reduction in AHI with intervention, while two reported no significant differences. One of the included studies that reported improved AHI (Labarca et al.) only found this AHI reduction when comparing MAD plus mouth taping to MAD alone [9]. When looking into the patient subgroup with mild OSA (defined by AHI < 15), utilization of MAD or MAD plus mouth taping did show significant reduction of AHI. When this study examined baseline AHI compared to mouth tape alone, there was no significant difference in AHI [9]. Other primary outcomes utilized by authors, such as snoring index, ODI, CPAP pressures, and mouth leak, showed significant differences favoring oral occluding strategies, however, many of these primary outcomes were shown in two or fewer studies. The clinical significance of these differences is also unclear, as none represents a gold standard metric of OSA severity.

Lee et al., Huang et al., and Labarca et al. were three of the six studies showing a level of reduction in AHI with oral occluding devices [2,4,9]. However, when looking closer into these studies, Lee et al. and Huang et al. only included individuals with AHIs of less than 15 [2,4]. The classification varies between studies, however, classically, an AHI of 5–15 is considered mild OSA [18]. The AHI improvement in the study by Lee et al. was 8.3 to 4.7 (mild to borderline mild), and 12 to 7.8 in the study by Huang et al (mild to persistently mild). With the criticism of AHI as a marker for disease severity in OSA, it is unclear whether these reported significant reductions in AHI are meaningful clinically [20,21]. Lee et al. and Huang et al., were able to report a significant reduction of SI alongside this AHI reduction but no other clinical factors or symptoms can be commented on. Labarca et al. included a larger range of individuals with AHIs from 10–50 [9]. However, their study only reported significant reductions in AHI for those individuals that were utilizing a MAD plus mouth tape compared to those with MAD alone, while comparing oral tape to baseline AHI showed no significant difference [9]. Although, when looking at subgroup analysis in the study by Labarca et al., those with mild OSA (AHI > 15), did have significant improvement in AHI with MAD or MAD plus mouth taping [9]. These three studies also excluded any individuals with any form of nasal obstruction including allergic rhinitis, chronic rhinitis, septal deviation, sinonasal disease as well as tonsils of grade three or above [2,4,9]. Therefore, it would be fair to assume that of the patients selected for these studies, any form of oral occlusion, would allow them to continue to breathe comfortably through their nose when asleep. However, the danger arises with the trend of mouth taping in those individuals who sleep with their mouths open when baseline nasal obstruction or nasal pathology is an underlying reason. Lee at al., also mention in their discussion that mouth taping is not recommended in patients with moderate to severe OSA as it may impose dangers rather than benefits in these groups of patients [4]. Huang et al. 2015, also discuss that the safety or efficacy of oral occlusion/taping cannot be elucidated from their study given the single small institution case series without a control group [2].

With respect to risks of mouth taping, there was explicit discussion in four out of ten of the studies indicating that oral occlusion either through taping, sealing, or chin strapping could pose a serious risk of asphyxiation in the presence of nasal obstruction or regurgitation (Table 2) [2,4,13,16]. Therefore, the social media phenomena of mouth taping as a means to stop mouth breathing would seem to be guided by poor evidence and can even lead to risk of detrimental effects in individuals with serious nasal obstruction as a cause of oral breathing.

Other primary outcomes measured by three different studies were anatomic measurements by Madeiro et al., Bachour et al., and Huang et al. All three studies demonstrated that with ceasing oral breathing, oropharynx spacing increased significantly [2,13,14]. These findings align with the study by Hsu et al. 2021, who assessed patients undergoing drug-induced sleep endoscopy (DISE) and saw that oral breathing was associated with a higher degree and prevalence of lateral pharyngeal and tongue base collapse [22]. Hsu et al. 2021, theorize that the mechanism behind this is three part – the first arm is the association of mouth breathing and higher upper airway resistance compared to nasal breathing, leading to increased obstructive apneas and hypoapneas [23]. The second arm is that mouth breathing decreases airway mucosa moisture and increases oropharyngeal wall surface tension causing difficulty reopening the upper airway [24]. The third arm is that nasal breathing activates nasal receptors that maintain spontaneous ventilation and oropharyngeal muscle tone which is also important for genioglossal activity [22,25]. However, both Bachour et al., and Huang et al. used cephalometric radiography in awake patients lying supine for their anatomical measurements [2,13]. Madiero et al. was the only study that assessed the oropharynx anatomy while patients were asleep using a pediatric bronchoscope [14].

Yang et al., similarly, studied patients with OSA undergoing DISE to assess total inspiratory flow in open and closed mouth settings [26]. Their group looked at 54 patients with a median AHI of 26.9 and median BMI of 28.9. Their study found that for their 32 patients with moderate levels of mouth breathing (oral airflow equating to 0.05–2.20 L/min), mouth closure increased inspiratory airflow by 2.0L/min. For their 10 patients with near-zero mouth breathing (<0.05 L/min), mouth closure had no significant change to inspiratory airflow (0.9 L/min). For their 12 patients with high levels of mouth breathing (>2.2 L/min), mouth closure was actually detrimental and decreased airflow by -1.86 L/min [26]. When assessing the anatomy of patients, upstream collapse was associated with greater mouth breathing and a negative response to mouth closure, especially with anteroposterior velum/soft palate collapse and concentric soft palate collapse [26]. These findings again seem to corroborate with the findings with Madeiro et al., Bachour et al., that oropharynx spacing and airflow volume increases when the mouth is closed during sleep [13,14,26]. Furthermore, Azarbarzin et al., were able to demonstrate in individuals with OSA, palatal prolapse into the velo/nasopharynx and causing expiratory flow limitation and a compensatory shunting of airflow through the mouth [27]. Taken all together, this could provide additional explanation as to why the mouth puffing phenomenon was found in the two studies by Jau et al., and that those with palatal collapse/prolapse would not benefit from mouth taping [1,12,27]. Therefore, for specific patient populations there is upstream or soft palate obstruction, mouth taping would appear to be an ineffective treatment. Additionally, Yang et al., have shown that for certain patients, namely those with high baseline mouth breathing during sleep and palatal obstruction, there are detrimental effects with forced mouth closure and that forced mouth closure during sleep is not universally beneficial [26].

The major limitations of our study include the heterogeneity of our studies, the poor quality of data based on the Newcastle-Ottawa Scale, and the limited number of studies. While we tried to make meaningful interpretations and conclusions with the small number of studies, it remains a fact that the results were very heterogenous and unfortunately statistical analysis could not be performed. Therefore, there needs to be more studies and higher quality studies to provide conclusive evidence of the safety and efficacy of this practice.

Overall, the summative data from the identified studies of this systematic review do not lend strong support to the idea of utilizing mouth taping or other occlusive devices for the improvement of OSA. All studies were of poor quality for different reasons as per the Newcastle-Ottawa assessment scale. Therefore, taking all the data together, there does seem to be a very specific use-case scenario in patient population where OSA is mild that mouth taping or occlusion may improve AHI. However, in other patient populations with nasal obstruction as a cause of mouth breathing or more severe forms of OSA, there is little evidence to support any clinical benefit for this practice. Moreover, the data identifies potential risk associated with the practice of oral occlusion for mouth breathing, SBD, or OSA.

## Conclusion

This systematic review reviewed 10 studies looking into different forms of oral occlusion in the setting of OSA or mouth breathing. Some studies report very minor improvement in certain outcomes such as AHI, ODI, and snoring index. However, the evidence for mouth taping as a treatment modality for mouth breathing, OSA, or SDB is minimal in most patient population groups outside of mild OSA, and not clinically signficiant. Moreover, there are potential serious detrimental health outcomes to those with nasal obstruction who seek oral taping as means to ameliorate their mouth breathing, OSA, or SDB during sleep. The existing data does not support mouth taping or oral occlusion as a sound clinical intervention for the general population with sleep disordered breathing.

## Supporting information

S1 Fig Search strategy utilized for the systematic review. (DOCX)

## References

[1] Jau J-Y, Kuo TBJ, Li LPH, Chen T-Y, Lai C-T, Huang P-H, et al. Mouth puffing phenomena of patients with obstructive sleep apnea when mouth-taped: device’s efficacy confirmed with physical video observation. Sleep Breath. 2023;27:153–64. doi: 10.1007/s11325-022-02588-0 35277783 PMC9992075

[2] Huang T-W, Young T-H. Novel porous oral patches for patients with mild obstructive sleep apnea and mouth breathing: a pilot study. Otolaryngol Head Neck Surg. 2015;152(2):369–73. doi: 10.1177/0194599814559383 25450408

[3] Ahn YM. Treatment of obstructive sleep apnea in children. Korean J Pediatr. 2010;53(10):872–9. doi: 10.3345/kjp.2010.53.10.872 21189957 PMC3004500

[4] Lee Y-C, Lu C-T, Cheng W-N, Li H-Y. The impact of mouth-taping in mouth-breathers with mild obstructive sleep apnea: a preliminary study. Healthcare (Basel). 2022;10(9):1755. doi: 10.3390/healthcare10091755 36141367 PMC9498537

[5] Valera FCP, Travitzki LVV, Mattar SEM, Matsumoto MAN, Elias AM, Anselmo-Lima WT. Muscular, functional and orthodontic changes in pre school children with enlarged adenoids and tonsils. Int J Pediatr Otorhinolaryngol. 2003;67(7):761–70. doi: 10.1016/s0165-5876(03)00095-8 12791452

[6] Rappai M, Collop N, Kemp S, deShazo R. The nose and sleep-disordered breathing: what we know and what we do not know. Chest. 2003;124(6):2309–23. doi: 10.1378/chest.124.6.2309 14665515

[7] Yi-Fong Su V, Chou K-T, Tseng C-H, Kuo C-Y, Su K-C, Perng D-W, et al. Mouth opening/breathing is common in sleep apnea and linked to more nocturnal water loss. Biomed J. 2023;46(3):100536. doi: 10.1016/j.bj.2022.05.001 35552020 PMC10209680

[8] Jung J-Y, Kang C-K. Investigation on the effect of oral breathing on cognitive activity using functional brain imaging. Healthcare (Basel). 2021;9(6):645. doi: 10.3390/healthcare9060645 34072444 PMC8228257

[9] Labarca G, Sands SA, Cohn V, Demko G, Vena D, Messineo L, et al. Mouth closing to improve the efficacy of mandibular advancement devices in sleep apnea. Ann Am Thorac Soc. 2022;19(7):1185–92. doi: 10.1513/AnnalsATS.202109-1050OC 35254967

[10] Osman AM, Toson B, Naik GR, Mukherjee S, Delbeck M, Hahn M, et al. A novel TASK channel antagonist nasal spray reduces sleep apnea severity in physiological responders: a randomized, blinded, trial. Am J Physiol Heart Circ Physiol. 2024;326(3):H715–23. doi: 10.1152/ajpheart.00541.2023 38214905

[11] Bhat S, Gushway-Henry N, Polos PG, DeBari VA, Riar S, Gupta D, et al. The efficacy of a chinstrap in treating sleep disordered breathing and snoring. J Clin Sleep Med. 2014;10(8):887–92. doi: 10.5664/jcsm.3962 25126035 PMC4106943

[12] Jau J-Y, Kuo TBJ, Li LPH, Chen T-Y, Hsu Y-S, Lai C-T, et al. Mouth puffing phenomenon and upper airway features may be used to predict the severity of obstructive sleep apnea. Nat Sci Sleep. 2023;15:165–74. doi: 10.2147/NSS.S384387 37032816 PMC10081528

[13] Bachour A, Hurmerinta K, Maasilta P. Mouth closing device (chinstrap) reduces mouth leak during nasal CPAP. Sleep Med. 2004;5(3):261–7. doi: 10.1016/j.sleep.2003.11.004 15165532

[14] Madeiro F, Andrade RGS, Piccin VS, Pinheiro GDL, Moriya HT, Genta PR, et al. Transmission of oral pressure compromises oronasal CPAP efficacy in the treatment of OSA. Chest. 2019;156: 1187–94. doi: 10.1016/j.chest.2019.05.024 31238041

[15] Rapoport DM. Methods to stabilize the upper airway using positive pressure. Sleep. 1996;19(9 Suppl):S123-30. doi: 10.1093/sleep/19.suppl_9.s123 9122569

[16] Teschler H, Stampa J, Ragette R, Konietzko N, Berthon-Jones M. Effect of mouth leak on effectiveness of nasal bilevel ventilatory assistance and sleep architecture. Eur Respir J. 1999;14(6):1251–7. doi: 10.1183/09031936.99.14612519 10624751

[17] Wells G, Shea B, O’Connell J. The Newcastle-Ottawa Scale (NOS) for Assessing The Quality of Nonrandomised Studies in Meta-analyses. Ottawa Heal Res Inst Web site. 2014;7.

[18] Duarte M, Pereira-Rodrigues P, Ferreira-Santos D. The role of novel digital clinical tools in the screening or diagnosis of obstructive sleep apnea: systematic review. J Med Internet Res. 2023;25:e47735. doi: 10.2196/47735 37494079 PMC10413091

[19] Malhotra A, Ayappa I, Ayas N, Collop N, Kirsch D, Mcardle N, et al. Metrics of sleep apnea severity: beyond the apnea-hypopnea index. Sleep. 2021;44(7):zsab030. doi: 10.1093/sleep/zsab030 33693939 PMC8271129

[20] Pevernagie DA, Gnidovec-Strazisar B, Grote L, Heinzer R, McNicholas WT, Penzel T, et al. On the rise and fall of the apnea-hypopnea index: a historical review and critical appraisal. J Sleep Res. 2020;29(4):e13066. doi: 10.1111/jsr.13066 32406974

[21] Won CHJ. When will we ditch the AHI? J Clin Sleep Med. 2020;16(7):1001–3. doi: 10.5664/jcsm.8594 32441250 PMC7954058

[22] Hsu Y-B, Lan M-Y, Huang Y-C, Kao M-C, Lan M-C. Association between breathing route, oxygen desaturation, and upper airway morphology. Laryngoscope. 2021;131(2):E659–64. doi: 10.1002/lary.28774 32473063

[23] Fitzpatrick MF, McLean H, Urton AM, Tan A, O’Donnell D, Driver HS. Effect of nasal or oral breathing route on upper airway resistance during sleep. Eur Respir J. 2003;22(5):827–32. doi: 10.1183/09031936.03.00047903 14621092

[24] Verma M, Seto-Poon M, Wheatley JR, Amis TC, Kirkness JP. Influence of breathing route on upper airway lining liquid surface tension in humans. J Physiol. 2006;574(Pt 3):859–66. doi: 10.1113/jphysiol.2005.102129 16690717 PMC1817732

[25] Basner RC, Simon PM, Schwartzstein RM, Weinberger SE, Weiss JW. Breathing route influences upper airway muscle activity in awake normal adults. J Appl Physiol (1985). 1989;66(4):1766–71. doi: 10.1152/jappl.1989.66.4.1766 2732169

[26] Yang H, Huyett P, Wang T-Y, Sumner J, Azarbarzin A, Labarca GPT, et al. Mouth closure and airflow in patients with obstructive sleep apnea: a nonrandomized clinical trial. JAMA Otolaryngol Head Neck Surg. 2024;150(11):1012–9. doi: 10.1001/jamaoto.2024.3319 39361293 PMC12316187

[27] Azarbarzin A, Sands SA, Marques M, Genta PR, Taranto-Montemurro L, Messineo L, et al. Palatal prolapse as a signature of expiratory flow limitation and inspiratory palatal collapse in patients with obstructive sleep apnoea. Eur Respir J. 2018;51(2):1701419. doi: 10.1183/13993003.01419-2017 29444914 PMC5915321

