# Supplementary material for: Breaking social media fads and uncovering the safety and efficacy of mouth taping in patients with mouth breathing, sleep disordered breathing, or obstructive sleep apnea: A systematic review
Source: PLoS One. 2025 May 21;20(5):e0323643. doi: 10.1371/journal.pone.0323643 (PMC12094774; doi:10.1371/journal.pone.0323643)
Supplement: S1 Fig — (DOCX) [file pone.0323643.s001.docx]

**Supplementary Figure 1: Search Strategy Utilized for The Systematic Review**

Database: Ovid MEDLINE(R) ALL <1946 to February 13, 2024>

Search Strategy:

1 *Surgical Tape/ and (mouth$1 or mouthpiece$ or open-mouth$ or oral$1).tw,kf. (12)

2 ((mouth$1 or mouthpiece$ or open-mouth$ or oral$1 or lip or lips) adj3 (adhesive$ or patch$ or tape or taped or taping$ or sealed or sealing$ or seal)).tw,kf,kw. or chinstrap$.tw,kf. (1216)

3 or/1-2 (1227)

4 exp Sleep/ (100978)

5 sleep$.mp. or night$.ti. or night$.ab. /freq=3 (288356)

6 or/4-5 (292186)

7 3 and 6 (50)

8 Mouth Breathing/ or breath$.mp. (163247)

9 3 and 6 and 8 (25)

10 (hazard$ or unsafe or safety or harm$ or complication$ or poison$ or risk$).mp. or AE.fs. or MO.fs. or PO.fs. or TO.fs. or CT.fs. or side-effect$.mp. or (undesirable adj1 effect$).mp. or (treatment adj1 emergent).mp. or tolerab$.mp. or toxic$.mp. or adrs.mp. or (adverse adj2 (effect or effects or reaction or reactions or event or events or outcome or outcomes)).mp. [FILTER MEDLINE Adverse Effects ] (9482228)

11 3 and 8 and 10 (27)

12 9 or 11 (47)

Database: Embase Classic+Embase <1947 to 2024 February 13>

Search Strategy:

1 mouth taping/ or adhesive mouthpiece/ or porous oral patch/ [Candidate Terms ] (3)

2 "masks, noses and mouth pieces"/ or breathing mouthpiece/ (272)

3 ((mouth$1 or mouthpiece$ or open-mouth$ or oral$1 or lip or lips) adj3 (adhesive$ or patch$ or tape or taped or taping$ or sealed or sealing$ or seal)).tw,kf,kw. or chinstrap$.tw,kw. (1737)

4 or/1-3 (2003)

5 sleep/ or microsleep/ or night sleep/ or exp sleep debt/ or sleep pattern/ or sleep quality/ or exp sleep stage/ or sleep time/ or sleep waking cycle/ or exp sleep disorder assessment/ (237417)

6 sleep$.tw,kw. or night$.ti. or night$.ab. /freq=3 (398766)

7 or/5-6 (430552)

8 4 and 7 (97)

9 exp *breathing/ or mouth breathing/ or breath$.tw,kw. (290272)

10 4 and 7 and 9 (37)

11 (hazard$ or unsafe or safety or harm$ or complication$ or poison$ or risk$).tw,kw. or AE.fs. or pv.fs. or side-effect$.tw,kw. or (undesirable adj1 effect$).tw,kw. or (treatment adj1 emergent).tw,kw. or tolerab$.tw,kw. or toxic$.tw,kw. or adrs.tw,kw. or (adverse adj2 (effect or effects or reaction or reactions or event or events or outcome or outcomes)).tw,kw. [FILTER EMBASE Adverse Effects ] (9330437)

12 4 and 9 and 11 (40)

13 10 or 12 (73)
