# Supplementary material for: Breaking social media fads and uncovering the safety and efficacy of mouth taping in patients with mouth breathing, sleep disordered breathing, or obstructive sleep apnea: A systematic review
Source: PLoS One. 2025 May 21;20(5):e0323643. doi: 10.1371/journal.pone.0323643 (PMC12094774; doi:10.1371/journal.pone.0323643)
Supplement: Attachment — Submitted filename: Review Comments v 1.2.docx [file pone.0323643.s002.docx]

# Review Comments

# Summary

This systematic review evaluates the efficacy and safety of mouth taping for addressing mouth breathing, sleep-disordered breathing (SDB), and obstructive sleep apnea (OSA). It explores a growing trend popularized on social media and synthesizes the available evidence on its potential risks and benefits. While the review highlights critical concerns and provides valuable insights into current research, there are limitations in comparative insights, heterogeneity of included studies, and consideration of recent key publications.

# Strengths

The manuscript has several notable strengths. First, it addresses a timely and highly relevant public health concern by analyzing the safety and efficacy of mouth taping, a practice that has gained widespread popularity despite the lack of substantial scientific validation. Second, the authors have employed a systematic methodology, adhering to PRISMA guidelines and utilizing a structured, librarian-assisted search strategy. This approach ensures comprehensive coverage of both indexed and non-indexed studies, enhancing the methodological rigor of the review. Third, the manuscript effectively highlights potential risks, such as asphyxiation and complications in individuals with nasal obstruction, which is a critical clinical consideration for practitioners who may encounter patient inquiries about this intervention. Lastly, the use of the Newcastle-Ottawa Scale to evaluate the included studies provides readers with a clear understanding of the quality and limitations of the evidence, further strengthening the manuscript's overall credibility..

# Major concerns

## C1. Lack of Comparative Insights:

The review misses the opportunity to compare its findings with recent studies, such as the *JAMA Otolaryngology* paper (*Mouth Closure and Airflow in Patients With Obstructive Sleep Apnea: A Nonrandomized Clinical Trial*). This study provides critical insights into how mouth closure influences airflow and sleep outcomes in controlled settings. Integrating these findings would enhance the depth and relevance of the discussion.

## C2. Heterogeneity in Included Studies:

The studies included in the review vary widely in methodology, patient populations, and outcome measures. While this is noted, the conclusion that mouth taping is ineffective is not fully justified given this heterogeneity. Subgroup analyses or meta-analytical techniques could offer more nuanced conclusions. For example, Studies showing significant AHI reductions primarily included patients with mild OSA (baseline AHI < 15), such as:

- **Lee et al.**: Baseline AHI = 8.3, reduced to 4.7.
- **Huang et al.**: Baseline AHI = 12, reduced to 7.8.

These results suggest that mouth taping may be effective for mild OSA but less so for moderate-to-severe cases. The review should emphasize this distinction and explore other studies' baseline characteristics to better contextualize the findings.

## C3. Recent Research Not Included:

Recent studies, such as the Harvard group's work on rostral airway obstruction and its influence on mouth taping efficacy, could significantly enhance the review (*JAMA Otolaryngol Head Neck Surg.* 2024;150(11):1012-1019. doi:10.1001/jamaoto.2024.3319). Including these findings would position the manuscript as a landmark paper in this field, reframing mouth taping as a targeted intervention rather than a one-size-fits-all solution.

# Comments on the Results Presentation

## The manuscript mentions: Jau et al. This group assessed and validated a “mouth puffing” device while patients’ mouths were taped. Jau et al. define mouth puffing as a derivation of mouth breathing while patients’ mouths are occluded with tape. As such, they utilized accelerometers on the sides of both cheeks to detect the mouth puffing phenomenon while patients’ mouths were occluded with tape1. They found that AHI was significantly reduced in individuals who had no mouth puffing compared to both side or complete mouth puffing

C4. In relation to the manuscript, could it be that "mouth puffing," as described by Jau et al., actually represents patients with palatal prolapse and expiratory flow limitation?

## The manuscript mentions: Arousal index was assessed by Osman et al. Patients in the novel spray plus tape group compared to placebo had a significantly reduced arousal index with reduced events per hour.

C5. This outcome could potentially reflect the effect of mouth taping; however, given that this is a drug study, it seems more likely that the drug itself had a greater impact on the results. Wouldn’t this interpretation be more consistent with the study design?

## The manuscript mentions: Bhat et al. also demonstrated that REM sleep percentage was significantly lower with patients using chinstraps compared to those on optimal CPAP12. Total sleep time was also significantly lower with chinstrap compared to patients during their diagnostic PSG study12 The cited reduction in REM sleep percentage and total sleep time (TST) with chin straps should be interpreted cautiously, as the original study only used chin straps for the first two hours of a split-night PSG protocol:

C3. The original Bhat study explicitly states: "Polysomnogram [PSG] underwent a modified split-night PSG, using only a chinstrap for the first 2 hours of sleep, followed by CPAP titration for the remainder of the night." This indicates that the diagnostic PSG was conducted as a full-night study, while the chinstrap was only used during the first 2 hours of the CPAP titration night. Given this setup, wouldn't it be inevitable for the total sleep time (TST) to be shorter during the chinstrap period and, consequently, the REM sleep percentage to differ?

# Comments on the Discussion section

## The manuscript mentions: Lee et al., Huang et al., and Labarca et al. were three of the six studies showing a level of reduction in AHI with oral occluding devices2,4,10. However, when looking closer into these studies, Lee et al. and Huang et al. only included individuals with AHIs of less than 152,4. The classification varies between studies, however, classically, an AHI of 5-15 is considered mild OSA18. The AHI improvement in the study by Lee et al. was 8.3 to 4.7 (mild to borderline mild), and 12 to 7.8 in the study by Huang et al (mild to persistently mild). With the criticism of AHI as a marker for disease severity in OSA, it is unclear whether these reported significant reductions in AHI are meaningful clinically20,21.

C4. As the authors demonstrated in the primary outcome section of the results, it cannot be denied that taping statistically lowers AHI in the entire cohort or specific patient subgroups. For instance, the Labarca study states: "A total of 21 participants were included (baseline AHI = 24.3 ± 9.9 events/h). The median AHI (interquartile range) in the MAD and MAD + AMT arms were 10.5 (5.4-19.6) events/h and 5.6 (2.2-11.7) events/h (P = 0.02), respectively. A total of 76% of individuals achieved an AHI of <10 events/h in the MAD + AMT arm versus 43% in the MAD arm (P < 0.01). Finally, the observed effect was similar in moderate to severe OSA (AHI ⩾ 15 events/h) in terms of absolute reduction and treatment responders, and AMT alone did not significantly reduce the AHI compared with baseline." This aligns with the authors' point that, in a randomized study, the combination of MAD + mouth tape (AMT) is more effective than MAD alone. This finding clearly demonstrates the effect of AMT in at least certain subgroups.

Additionally, “Jau et al. assessed and validated a ‘mouth puffing’ device while patients’ mouths were taped. Jau et al. define mouth puffing as a derivation of mouth breathing while patients’ mouths are occluded with tape. As such, they utilized accelerometers on the sides of both cheeks to detect the mouth puffing phenomenon while patients’ mouths were occluded with tape. They found that AHI was significantly reduced in individuals who had no mouth puffing compared to both side or complete mouth puffing.” This finding also suggests that mouth taping may worsen apnea in specific subgroups, particularly in patients with mouth puffing. Given that individuals with mouth puffing are likely to have palatal prolapse or expiratory flow limitation, wouldn’t it be reasonable to interpret this as evidence that mouth taping may be effective in subgroups without these anatomical or physiological features?

Additionally, considering that MAD and similar interventions are generally effective in patients with mild OSA, wouldn’t it be more appropriate to interpret this as evidence of effectiveness in mild patient populations, rather than stating, *“it is unclear whether these reported significant reductions in AHI are meaningful clinically”?* This perspective aligns better with the observed outcomes.

## The manuscript mentions: The third arm is that nasal breathing activates nasal receptors that maintain spontaneous ventilation and oropharyngeal muscle tone which is also important for genioglossal activity22.

C5. his section's reference should be clarified, as reference 22 appears to be related to DISE and not to nasal receptors as stated. Could you confirm the correct reference for this claim? It is important to ensure the accuracy of cited studies to maintain the validity of the discussion.

## The manuscript mentions: Other primary outcomes measured by three different studies were anatomic measurements by Madeiro et al., Bachour et al., and Huang et al.. All three studies demonstrated that with ceasing oral breathing, oropharynx spacing increased significantly2,14,15. These findings align with the study by Hsu et al. 2021, who assessed patients undergoing drug-induced sleep endoscopy and saw that oral breathing was associated with a higher degree and prevalence of lateral pharyngeal and tongue base collapse22. Hsu et al. 2021, theorize that the mechanism behind this is three part – the first arm is the association of mouth breathing and higher upper airway resistance compared to nasal breathing, leading to increased obstructive apneas and hypoapneas24. The second arm is that mouth breathing decreases airway mucosa moisture and increases oropharyngeal wall surface tension causing difficulty reopening the upper airway25. The third arm is that nasal breathing activates nasal receptors that maintain spontaneous ventilation and oropharyngeal muscle tone which is also important for genioglossal activity22. However, both Bachour et al., and Huang et al. used cephalometric radiography in awake patients lying supine for their anatomical measurements2,14. Madiero et al. was the only study that assessed the oropharynx anatomy while patients were asleep using a pediatric bronchoscope15.

C6. The conclusion in this section requires further consideration. For example, “Hsu et al. 2021, who assessed patients undergoing drug-induced sleep endoscopy and saw that oral breathing was associated with a higher degree and prevalence of lateral pharyngeal and tongue base collapse” suggests that mouth closure may have anatomical benefits. While this conclusion itself could be subject to error, it does not indicate negative effects of mouth taping. Moreover, the positive effects of mouth taping, as identified in references 2, 4, and 15, cannot be dismissed based on this evidence.

This section would benefit from a different approach. For instance, focusing on identifying subgroups of patients who benefit from mouth taping versus those who do not could lead to a more robust and clinically meaningful conclusion. Such an approach could significantly enhance the quality and relevance of the manuscript.

# Conclusion

The authors have conducted a thorough and systematic analysis of the relevant data, and I agree with their observation that there is no definitive evidence supporting the efficacy of mouth taping. However, based on the authors' analysis, it seems more accurate to conclude that the effects of mouth taping are inconsistent rather than absent.

To make this manuscript more impactful, shifting the focus from merely evaluating the overall efficacy of mouth taping to other aspects might be beneficial. For example, the authors could consider summarizing the varying effects of mouth taping, proposing potential indication groups, or systematically discussing the *Potential Complications* outlined in Table 2. Such an approach could provide more clinical relevance and guidance, enhancing the manuscript’s contribution to the field.

# Final Recommendation

**Major Revisions.**
This manuscript has potential but requires significant revisions to incorporate recent research, refine its conclusions, and provide actionable guidance for clinicians. By addressing these issues, the manuscript could become a key resource in discussing mouth taping and related interventions for OSA.
