# Supplementary material for: Breaking social media fads and uncovering the safety and efficacy of mouth taping in patients with mouth breathing, sleep disordered breathing, or obstructive sleep apnea: A systematic review
Source: PLoS One. 2025 May 21;20(5):e0323643. doi: 10.1371/journal.pone.0323643 (PMC12094774; doi:10.1371/journal.pone.0323643)
Supplement: Attachment — Submitted filename: Review Comments Feb 11 2026.docx [file pone.0323643.s003.docx]

# Review Comments

# Summary

This systematic review evaluates the efficacy and safety of mouth taping for addressing mouth breathing, sleep-disordered breathing (SDB), and obstructive sleep apnea (OSA). It explores a growing trend popularized on social media and synthesizes the available evidence on its potential risks and benefits. While the review highlights critical concerns and provides valuable insights into current research, there are limitations in comparative insights, heterogeneity of included studies, and consideration of recent key publications.

# Strengths

The manuscript has several notable strengths. First, it addresses a timely and highly relevant public health concern by analyzing the safety and efficacy of mouth taping, a practice that has gained widespread popularity despite the lack of substantial scientific validation. Second, the authors have employed a systematic methodology, adhering to PRISMA guidelines and utilizing a structured, librarian-assisted search strategy. This approach ensures comprehensive coverage of both indexed and non-indexed studies, enhancing the methodological rigor of the review. Third, the manuscript effectively highlights potential risks, such as asphyxiation and complications in individuals with nasal obstruction, which is a critical clinical consideration for practitioners who may encounter patient inquiries about this intervention. Lastly, the use of the Newcastle-Ottawa Scale to evaluate the included studies provides readers with a clear understanding of the quality and limitations of the evidence, further strengthening the manuscript's overall credibility..

# Major concerns

## C1. Lack of Comparative Insights:

The review misses the opportunity to compare its findings with recent studies, such as the *JAMA Otolaryngology* paper (*Mouth Closure and Airflow in Patients With Obstructive Sleep Apnea: A Nonrandomized Clinical Trial*). This study provides critical insights into how mouth closure influences airflow and sleep outcomes in controlled settings. Integrating these findings would enhance the depth and relevance of the discussion.

This paper has been incorporated into our discussion section to compare our findings. (Page 16)

**Page 16:**
“Yang et al., similarly, studied patients with OSA undergoing DISE to assess total inspiratory flow in open and closed mouth settings^26^. Their group looked at 54 patients with a median AHI of 26.9 and median BMI of 28.9. Their study found that for their 32 patients with moderate levels of mouth breathing (oral airflow equating to 0.05-2.20 L/min), mouth closure increased inspiratory airflow by 2.0L/min. For their 10 patients with near-zero mouth breathing (<0.05 L/min), mouth closure had no significant change to inspiratory airflow (0.9 L/min). For their 12 patients with high levels of mouth breathing (>2.2 L/min), mouth closure was actually detrimental and decreased airflow by -1.86 L/min^26^. When assessing the anatomy of patients, upstream collapse was associated with greater mouth breathing and a negative response to mouth closure, especially with anteroposterior velum/soft palate collapse and concentric soft palate collapse^26^. These findings again seem to corroborate with the findings with Madeiro et al., Bachour et al., that oropharynx spacing and airflow volume increases when the mouth is closed during sleep^13,14,26^. Therefore, for specific patient populations where AHI is mild and there is limited upstream or soft palate obstruction, there does seem to be a potential for benefit. However, interestingly, Yang et al., have shown that for certain patients, namely those with high baseline mouth breathing during sleep, there are detrimental effects with forced mouth closure and that forced mouth closure during sleep is not universally beneficial^26^.”

## C2. Heterogeneity in Included Studies:

The studies included in the review vary widely in methodology, patient populations, and outcome measures. While this is noted, the conclusion that mouth taping is ineffective is not fully justified given this heterogeneity. Subgroup analyses or meta-analytical techniques could offer more nuanced conclusions. For example, Studies showing significant AHI reductions primarily included patients with mild OSA (baseline AHI < 15), such as:

- **Lee et al.**: Baseline AHI = 8.3, reduced to 4.7.
- **Huang et al.**: Baseline AHI = 12, reduced to 7.8.

These results suggest that mouth taping may be effective for mild OSA but less so for moderate-to-severe cases. The review should emphasize this distinction and explore other studies' baseline characteristics to better contextualize the findings.

## Dear Reviewers,

## We were not able to perform a meta-analysis for this paper for the following reasons:

## 1. The heterogeneity of the available data implies that studies are different, and combining such disparate data can lead to a meaningless or misleading result, as the studies may not be addressing the same underlying question or outcome.

## 2. The studies we reviewed had variations in populations introducing confounding factors that can obscure the true effect of the intervention being studied.

## 3. The different studies reviewed had various subgroup analyses such that a meta-analysis could mask important subgroup effects. These subgroup effects might be missed in a meta-analysis, leading to a misleading conclusion about the overall effectiveness of the intervention.

## 4. The results of a meta-analysis are only generalizable to the population of studies included in the analysis. When studies are heterogeneous like on our paper, the results might not be generalizable to any specific population, limiting the practical implications of the findings.

**Our Statistician has also provided us with detailed insight as well for the reviewers:** Despite the numbers described above, a thorough review of each of these studies’ characteristics depicts the fragility of the above-mentioned results, which leads to low external validity and questionable clinical relevance. Given the considerable number of biases in both studies, pooling these studies' data would lead to a low-powered conclusion, with a potentially misleading scientific conclusion negatively impacting this patient population.

The study of Huang et al. claims that a convenience sample of 30 patients using POP presented a significant median reduction in AHI of 4.2 (12 to 7.8) without mentioning any degree of dispersion (i.e., IQR, Range, etc.). Also, the absence of a control group and an effect size estimate reduces its validity, statistical power, and interpretation potential. Although a meta-analysis can still be performed between studies reporting medians, using data transformation to approximate means and standard deviations (SDs), we would still need interquartile ranges (IQRs) or medians and ranges to perform such analysis.

Meanwhile, the study of Lee et al. reports a median reduction in AHI of 3.6. The results presented (Mouth breathing Median 8.3, IQR 6.2–12.9; Nasal breathing Median 4.7, IQR 2.7–7.6) indicate a large interquartile range (IQR) in both groups relative to their medians, suggesting high variability within the dataset. Given the small sample size (n=20), this variability raises several critical concerns when the risk of outliers disproportionately influencing the median increases, particularly in small, skewed datasets, is significant. Methodological flaws, such as lack of sample size calculation and no randomization of the sequence of assessments (nasal and mouth breathing), associated with the wide IQR in both groups, also reduce the room for further conclusive analysis within this study.

Therefore, considering the available data, there are insufficient resources for a subgroup analysis or a meta-analysis. We expect that by addressing the gaps in the literature, the current study will foster the development of more robust and clinically applicable evidence while preventing underrepresented patient populations, such as patients with allergic rhinitis and chronic rhinosinusitis, and septal deviation, from exposing themselves to interventions with unproven efficacy and safety.

## C3. Recent Research Not Included:

Recent studies, such as the Harvard group's work on rostral airway obstruction and its influence on mouth taping efficacy, could significantly enhance the review (*JAMA Otolaryngol Head Neck Surg.* 2024;150(11):1012-1019. - doi:10.1001/jamaoto.2024.3319). Including these findings would position the manuscript as a landmark paper in this field, reframing mouth taping as a targeted intervention rather than a one-size-fits-all solution.

This paper has been added and incorporated into our manuscript. (Page 16)

**Page 16:**

“Yang et al., similarly, studied patients with OSA undergoing DISE to assess total inspiratory flow in open and closed mouth settings^26^. Their group looked at 54 patients with a median AHI of 26.9 and median BMI of 28.9. Their study found that for their 32 patients with moderate levels of mouth breathing (oral airflow equating to 0.05-2.20 L/min), mouth closure increased inspiratory airflow by 2.0L/min. For their 10 patients with near-zero mouth breathing (<0.05 L/min), mouth closure had no significant change to inspiratory airflow (0.9 L/min). For their 12 patients with high levels of mouth breathing (>2.2 L/min), mouth closure was actually detrimental and decreased airflow by -1.86 L/min^26^. When assessing the anatomy of patients, upstream collapse was associated with greater mouth breathing and a negative response to mouth closure, especially with anteroposterior velum/soft palate collapse and concentric soft palate collapse^26^. These findings again seem to corroborate with the findings with Madeiro et al., Bachour et al., that oropharynx spacing and airflow volume increases when the mouth is closed during sleep^13,14,26^. Therefore, for specific patient populations where AHI is mild and there is limited upstream or soft palate obstruction, there does seem to be a potential for benefit. However, interestingly, Yang et al., have shown that for certain patients, namely those with high baseline mouth breathing during sleep, there are detrimental effects with forced mouth closure and that forced mouth closure during sleep is not universally beneficial^26^.”

# Comments on the Results Presentation

## The manuscript mentions: Jau et al. This group assessed and validated a “mouth puffing” device while patients’ mouths were taped. Jau et al. define mouth puffing as a derivation of mouth breathing while patients’ mouths are occluded with tape. As such, they utilized accelerometers on the sides of both cheeks to detect the mouth puffing phenomenon while patients’ mouths were occluded with tape1. They found that AHI was significantly reduced in individuals who had no mouth puffing compared to both side or complete mouth puffing

C4. In relation to the manuscript, could it be that "mouth puffing," as described by Jau et al., actually represents patients with palatal prolapse and expiratory flow limitation? – This is quite possible but both studies do not touch upon this in much detail. Although their second study do show some anatomical correlations, but they cannot make any conclusive statements such as this. However, this is quite a realistic possibility of mouth puffing being a consequence of palatal prolapse and expiratory flow limitation.

## The manuscript mentions: Arousal index was assessed by Osman et al. Patients in the novel spray plus tape group compared to placebo had a significantly reduced arousal index with reduced events per hour.

C5. This outcome could potentially reflect the effect of mouth taping; however, given that this is a drug study, it seems more likely that the drug itself had a greater impact on the results. Wouldn’t this interpretation be more consistent with the study design?

This has been revised and addressed in the manuscript. There was a mistake in that the study actually showed increased arousal threshold with tape + novel spray indicating actually that the spray was not as effective when mouth taped. The study does comment that this could be due to increased sleep drive secondary to OSA, especially when mouth taped. (Page 11)

**Page 11:**

“Arousal threshold, as defined by the maximum estimated ventilatory drive just before coirtical arousal during NREM (non-rapid eye movement sleep) respiratory events that resulted in arousal, was assessed by Osman et al. Patients in the novel upper airway muscle dilator spray plus tape group compared to placebo had a significantly increased arousal index with reduced events per hour^10^. The study does comment that the spray is an effective upper airway dilator, but the effects may be impaired when the mouth is taped, due to an increased sleep drive secondary to OSA^10^.“

## The manuscript mentions: Bhat et al. also demonstrated that REM sleep percentage was significantly lower with patients using chinstraps compared to those on optimal CPAP12. Total sleep time was also significantly lower with chinstrap compared to patients during their diagnostic PSG study12 The cited reduction in REM sleep percentage and total sleep time (TST) with chin straps should be interpreted cautiously, as the original study only used chin straps for the first two hours of a split-night PSG protocol:

C3. The original Bhat study explicitly states: "Polysomnogram [PSG] underwent a modified split-night PSG, using only a chinstrap for the first 2 hours of sleep, followed by CPAP titration for the remainder of the night." This indicates that the diagnostic PSG was conducted as a full-night study, while the chinstrap was only used during the first 2 hours of the CPAP titration night. Given this setup, wouldn't it be inevitable for the total sleep time (TST) to be shorter during the chinstrap period and, consequently, the REM sleep percentage to differ?

- These very valid points have been addressed in the manuscript (page 12). The study does indeed go on to state that TST was shorter as expected, however, the reduction in REM sleep was analyzed a percentage of the total sleep time with chin strapping sleep vs non chin-strapping sleep.

**Page 12:**

“Although this study only performed chin strapping for the first two hours, the reduction in REM sleep with chinstrap compared to PSG was analyzed as a percentage of the total sleep time^11^. Bhat et al. also demonstrated that REM sleep percentage was significantly lower with patients using chinstraps compared to those on optimal CPAP^11^. Total sleep time was also significantly lower with chinstrap compared to patients during their diagnostic PSG study, although this was an expected result as chin strapping was only used for the first two hours^11^. Total sleep time was unchanged when comparing chinstrap to those on optimal CPAP^11^. Bhat et al. also showed that there was no significant difference in SpO2 nadir after chinstrap usage^11^.”

# Comments on the Discussion section

## The manuscript mentions: Lee et al., Huang et al., and Labarca et al. were three of the six studies showing a level of reduction in AHI with oral occluding devices2,4,10. However, when looking closer into these studies, Lee et al. and Huang et al. only included individuals with AHIs of less than 152,4. The classification varies between studies, however, classically, an AHI of 5-15 is considered mild OSA18. The AHI improvement in the study by Lee et al. was 8.3 to 4.7 (mild to borderline mild), and 12 to 7.8 in the study by Huang et al (mild to persistently mild). With the criticism of AHI as a marker for disease severity in OSA, it is unclear whether these reported significant reductions in AHI are meaningful clinically20,21.

C4. As the authors demonstrated in the primary outcome section of the results, it cannot be denied that taping statistically lowers AHI in the entire cohort or specific patient subgroups. For instance, the Labarca study states: "A total of 21 participants were included (baseline AHI = 24.3 ± 9.9 events/h). The median AHI (interquartile range) in the MAD and MAD + AMT arms were 10.5 (5.4-19.6) events/h and 5.6 (2.2-11.7) events/h (P = 0.02), respectively. A total of 76% of individuals achieved an AHI of <10 events/h in the MAD + AMT arm versus 43% in the MAD arm (P < 0.01). Finally, the observed effect was similar in moderate to severe OSA (AHI ⩾ 15 events/h) in terms of absolute reduction and treatment responders, and AMT alone did not significantly reduce the AHI compared with baseline." This aligns with the authors' point that, in a randomized study, the combination of MAD + mouth tape (AMT) is more effective than MAD alone. This finding clearly demonstrates the effect of AMT in at least certain subgroups.

These points have been considered and appropriate changes have been incorporated into the manuscript to reflect that mouth tape does show significant benefit in specific sub-populations. (Page 13 and 14)

**Page 13:**

### “When looking into the patient subgroup with mild OSA (defined by AHI >15), utilization of MAD or MAD plus mouth taping did show significant reduction of AHI.”

### **Page 14:**

### “Although, when looking at subgroup analysis in the study by Labarca et al., those with mild OSA (AHI>15), did have significant improvement in AHI with MAD or MAD plus mouth taping^9^.”

Additionally, “Jau et al. assessed and validated a ‘mouth puffing’ device while patients’ mouths were taped. Jau et al. define mouth puffing as a derivation of mouth breathing while patients’ mouths are occluded with tape. As such, they utilized accelerometers on the sides of both cheeks to detect the mouth puffing phenomenon while patients’ mouths were occluded with tape. They found that AHI was significantly reduced in individuals who had no mouth puffing compared to both side or complete mouth puffing.” This finding also suggests that mouth taping may worsen apnea in specific subgroups, particularly in patients with mouth puffing. Given that individuals with mouth puffing are likely to have palatal prolapse or expiratory flow limitation, wouldn’t it be reasonable to interpret this as evidence that mouth taping may be effective in subgroups without these anatomical or physiological features?

### These comments have also been addressed in the manuscript. (Page 16 and 17)

**Page 16:**

“Therefore, for specific patient populations where AHI is mild and there is limited upstream or soft palate obstruction, there does seem to be a potential for benefit. However, interestingly, Yang et al., have shown that for certain patients, namely those with high baseline mouth breathing during sleep, there are detrimental effects with forced mouth closure and that forced mouth closure during sleep is not universally beneficial^26^.”

**Page 17:**

“Therefore, taking all the data together, there does seem to be a very specific use-case scenario in patient population where OSA is mild that mouth taping or occlusion may improve AHI. However, in other patient populations with nasal obstruction as a cause of mouth breathing or more severe forms of OSA, there is little evidence to support any clinical benefit for this practice.”

Additionally, considering that MAD and similar interventions are generally effective in patients with mild OSA, wouldn’t it be more appropriate to interpret this as evidence of effectiveness in mild patient populations, rather than stating, *“it is unclear whether these reported significant reductions in AHI are meaningful clinically”?* This perspective aligns better with the observed outcomes.

This has also been addressed to indicate potential benefit in sub-populations (page 16 and 17)

**Page 16:**

“Therefore, for specific patient populations where AHI is mild and there is limited upstream or soft palate obstruction, there does seem to be a potential for benefit. However, interestingly, Yang et al., have shown that for certain patients, namely those with high baseline mouth breathing during sleep, there are detrimental effects with forced mouth closure and that forced mouth closure during sleep is not universally beneficial^26^.”

**Page 17:**

## “Therefore, taking all the data together, there does seem to be a very specific use-case scenario in patient population where OSA is mild that mouth taping or occlusion may improve AHI. However, in other patient populations with nasal obstruction as a cause of mouth breathing or more severe forms The manuscript mentions: The third arm is that nasal breathing activates nasal receptors that maintain spontaneous ventilation and oropharyngeal muscle tone which is also important for genioglossal activity22.

C5. his section's reference should be clarified, as reference 22 appears to be related to DISE and not to nasal receptors as stated. Could you confirm the correct reference for this claim? It is important to ensure the accuracy of cited studies to maintain the validity of the discussion.

- The authors did indeed perform DISE but this is from their discussion as well where they discuss the possible arms to explain the findings from their results.

## The manuscript mentions: Other primary outcomes measured by three different studies were anatomic measurements by Madeiro et al., Bachour et al., and Huang et al.. All three studies demonstrated that with ceasing oral breathing, oropharynx spacing increased significantly2,14,15. These findings align with the study by Hsu et al. 2021, who assessed patients undergoing drug-induced sleep endoscopy and saw that oral breathing was associated with a higher degree and prevalence of lateral pharyngeal and tongue base collapse22. Hsu et al. 2021, theorize that the mechanism behind this is three part – the first arm is the association of mouth breathing and higher upper airway resistance compared to nasal breathing, leading to increased obstructive apneas and hypoapneas24. The second arm is that mouth breathing decreases airway mucosa moisture and increases oropharyngeal wall surface tension causing difficulty reopening the upper airway25. The third arm is that nasal breathing activates nasal receptors that maintain spontaneous ventilation and oropharyngeal muscle tone which is also important for genioglossal activity22. However, both Bachour et al., and Huang et al. used cephalometric radiography in awake patients lying supine for their anatomical measurements2,14. Madiero et al. was the only study that assessed the oropharynx anatomy while patients were asleep using a pediatric bronchoscope15.

C6. The conclusion in this section requires further consideration. For example, “Hsu et al. 2021, who assessed patients undergoing drug-induced sleep endoscopy and saw that oral breathing was associated with a higher degree and prevalence of lateral pharyngeal and tongue base collapse” suggests that mouth closure may have anatomical benefits. While this conclusion itself could be subject to error, it does not indicate negative effects of mouth taping. Moreover, the positive effects of mouth taping, as identified in references 2, 4, and 15, cannot be dismissed based on this evidence.

This section would benefit from a different approach. For instance, focusing on identifying subgroups of patients who benefit from mouth taping versus those who do not could lead to a more robust and clinically meaningful conclusion. Such an approach could significantly enhance the quality and relevance of the manuscript.

- These valid comments have been addressed to demonstrate the specific use-case scenarios of certain patient populations and potential benefits in these patients. (Page 16 and 17)

**Page 16:**

“Therefore, for specific patient populations where AHI is mild and there is limited upstream or soft palate obstruction, there does seem to be a potential for benefit. However, interestingly, Yang et al., have shown that for certain patients, namely those with high baseline mouth breathing during sleep, there are detrimental effects with forced mouth closure and that forced mouth closure during sleep is not universally beneficial^26^.”

**Page 17:**

“Therefore, taking all the data together, there does seem to be a very specific use-case scenario in patient population where OSA is mild that mouth taping or occlusion may improve AHI. However, in other patient populations with nasal obstruction as a cause of mouth breathing or more severe forms

# Conclusion

The authors have conducted a thorough and systematic analysis of the relevant data, and I agree with their observation that there is no definitive evidence supporting the efficacy of mouth taping. However, based on the authors' analysis, it seems more accurate to conclude that the effects of mouth taping are inconsistent rather than absent.

To make this manuscript more impactful, shifting the focus from merely evaluating the overall efficacy of mouth taping to other aspects might be beneficial. For example, the authors could consider summarizing the varying effects of mouth taping, proposing potential indication groups, or systematically discussing the *Potential Complications* outlined in Table 2. Such an approach could provide more clinical relevance and guidance, enhancing the manuscript’s contribution to the field.

# Final Recommendation

**Major Revisions.**
This manuscript has potential but requires significant revisions to incorporate recent research, refine its conclusions, and provide actionable guidance for clinicians. By addressing these issues, the manuscript could become a key resource in discussing mouth taping and related interventions for OSA.

Thank you reviewers for your time looking over our manuscript. All reviewers have brought up such valuable and insightful points. We are grateful for your consideration of our manuscript and welcome any further suggestions for the improvement of our manuscript.

Sincerely,

Jess Rhee
