# Supplementary material for: Breaking social media fads and uncovering the safety and efficacy of mouth taping in patients with mouth breathing, sleep disordered breathing, or obstructive sleep apnea: A systematic review
Source: PLoS One. 2025 May 21;20(5):e0323643. doi: 10.1371/journal.pone.0323643 (PMC12094774; doi:10.1371/journal.pone.0323643)
Supplement: Attachment — Submitted filename: Reviewer Comments on Revised Manuscript.docx [file pone.0323643.s004.docx]

# **Reviewer Comments on Revised Manuscript**

Dear Authors,

I appreciate the efforts you have made in revising the manuscript and addressing the comments provided. The revisions have strengthened the manuscript, but there are several points that require further clarification and modifications. Below are my comments on the revised version:

## C2. Heterogeneity in Included Studies

The heterogeneity of the included studies remains a crucial factor in interpreting the findings. I strongly recommend that this be explicitly addressed in the **limitations section** of the manuscript to ensure transparency regarding the variability across studies.

## C4. Expiratory Flow Limitation and Palatal Prolapse

It would be beneficial to mention the potential role of **expiratory flow limitation and palatal prolapse** in interpreting the results. This could provide further insight into why mouth puffing occurs and why certain patient populations may not benefit from mouth taping.

## Page 13: Correction of Mild OSA Definition

The statement on **Page 13** currently reads:
*"When looking into the patient subgroup with mild OSA (defined by AHI >15), utilization of MAD or MAD plus mouth taping did show significant reduction of AHI."*
However, **mild OSA is classically defined as AHI <15, not AHI >15.** This should be corrected to:
**"Mild OSA (defined by AHI <15)"**

## C4 (Page 16): Misinterpretation of Palatal Obstruction and Mouth Taping Effectiveness

The authors state:
*"Therefore, for specific patient populations where AHI is mild and there is limited upstream or soft palate obstruction, there does seem to be a potential for benefit. However, interestingly, Yang et al., have shown that for certain patients, namely those with high baseline mouth breathing during sleep, there are detrimental effects with forced mouth closure and that forced mouth closure during sleep is not universally beneficial."*

However, this interpretation **needs correction based on the anatomical and physiological context**:

- **Mouth puffing is strongly associated with palatal prolapse**, which leads to expiratory flow limitation. This means that mouth puffing occurs when there is **palatal airway obstruction** during exhalation.
- **Patients with palatal prolapse typically have a longer palate, leading to palatal anteroposterior (AP) obstruction.**
- **Yang et al.'s study also demonstrated that patients with palatal obstruction do not benefit from mouth taping.**

Thus, the findings from both studies are actually **consistent**, showing that **patients with upper airway (palatal) obstruction do not respond well to mouth taping.**

## Critical Error on Page 16:

- The phrase **"limited upstream or soft palate obstruction"** suggests that mouth taping is beneficial for such patients, which **is incorrect.**
- The correct interpretation is that **mouth taping is ineffective in patients with significant upstream or soft palate obstruction.**
- **This is a critical error that must be corrected.**

## C5. Validity of Referencing Non-Empirical Discussion Points

- If the statement in **C5** is not based on direct research findings but rather reflects the authors' interpretation in the discussion, then **citing it as a reference must be reconsidered.**
- Discussion-based opinions from the original study authors should not be treated as empirical evidence.
- Please ensure that references used are **derived from actual study findings rather than speculative discussion points.**

**Conclusion**

The revised manuscript has improved, but several point should be addressed to ensure the accuracy and scientific integrity of the findings.

Key revisions needed:

- **Address study heterogeneity in the limitations section**
- **Correct the definition of mild OSA (AHI <15) on Page 13**
- **Revise the interpretation of mouth puffing and palatal prolapse on Page 16**
- **Correct the misleading statement that "limited upstream or soft palate obstruction" improves with mouth taping**
- **Re-evaluate the appropriateness of citing discussion-based opinions as references in C5**

Once these changes are made, the manuscript will be in a much stronger position.

Best regards,
