# Supplementary material for: Breaking social media fads and uncovering the safety and efficacy of mouth taping in patients with mouth breathing, sleep disordered breathing, or obstructive sleep apnea: A systematic review
Source: PLoS One. 2025 May 21;20(5):e0323643. doi: 10.1371/journal.pone.0323643 (PMC12094774; doi:10.1371/journal.pone.0323643)
Supplement: Attachment — Submitted filename: Reviewer Comments on Revised Manuscript March 16 2025.docx [file pone.0323643.s005.docx]

# **Reviewer Comments on Revised Manuscript**

Dear Authors,

I appreciate the efforts you have made in revising the manuscript and addressing the comments provided. The revisions have strengthened the manuscript, but there are several points that require further clarification and modifications. Below are my comments on the revised version:

Thank you reviewers for your time with our manuscript. We appreciate all the effort into making this a finalized polished piece.

## C2. Heterogeneity in Included Studies

The heterogeneity of the included studies remains a crucial factor in interpreting the findings. I strongly recommend that this be explicitly addressed in the **limitations section** of the manuscript to ensure transparency regarding the variability across studies.

This has been addressed in page 17 of our manuscript:

“The major limitations of our study include the heterogeneity of our studies, the poor quality of data based on the Newcastle-Ottawa Scale, and the limited number of studies. While we tried to make meaningful interpretations and conclusions with the small number of studies, it remains a fact that the results were very heterogenous and unfortunately statistical analysis could not be performed. Therefore, there needs to be more studies and higher quality studies to provide conclusive evidence of the safety and efficacy of this practice.”

## C4. Expiratory Flow Limitation and Palatal Prolapse

It would be beneficial to mention the potential role of **expiratory flow limitation and palatal prolapse** in interpreting the results. This could provide further insight into why mouth puffing occurs and why certain patient populations may not benefit from mouth taping.

This has been addressed on pages 15-16:

## Furthermore, Azarbarzin et al., were able to demonstrate in individuals with OSA, palatal prolapse into the velo/nasopharynx and causing expiratory flow limitation and a compensatory shunting of airflow through the mouth^27^. Taken all together, this could provide additional explanation as to why the mouth puffing phenomenon was found in the two studies by Jau et al., and that those with palatal collapse/prolapse would not benefit from mouth taping^1,12,27^. Therefore, for specific patient populations there is upstream or soft palate obstruction, mouth taping would appear to be an ineffective treatment.

## Page 13: Correction of Mild OSA Definition

The statement on **Page 13** currently reads:
*"When looking into the patient subgroup with mild OSA (defined by AHI >15), utilization of MAD or MAD plus mouth taping did show significant reduction of AHI."*
However, **mild OSA is classically defined as AHI <15, not AHI >15.** This should be corrected to:
**"Mild OSA (defined by AHI <15)"**

This has been addressed on page 13:

“When looking into the patient subgroup with mild OSA (defined by AHI <15), utilization of MAD or MAD plus mouth taping did show significant reduction of AHI.”

## C4 (Page 16): Misinterpretation of Palatal Obstruction and Mouth Taping Effectiveness

The authors state:
*"Therefore, for specific patient populations where AHI is mild and there is limited upstream or soft palate obstruction, there does seem to be a potential for benefit. However, interestingly, Yang et al., have shown that for certain patients, namely those with high baseline mouth breathing during sleep, there are detrimental effects with forced mouth closure and that forced mouth closure during sleep is not universally beneficial."*

However, this interpretation **needs correction based on the anatomical and physiological context**:

- **Mouth puffing is strongly associated with palatal prolapse**, which leads to expiratory flow limitation. This means that mouth puffing occurs when there is **palatal airway obstruction** during exhalation.
- **Patients with palatal prolapse typically have a longer palate, leading to palatal anteroposterior (AP) obstruction.**
- **Yang et al.'s study also demonstrated that patients with palatal obstruction do not benefit from mouth taping.**

Thus, the findings from both studies are actually **consistent**, showing that **patients with upper airway (palatal) obstruction do not respond well to mouth taping.**

## Thanking you reviewers for pointing out this misinterpretation. It has been revised and a study to tie concepts of mouth puffing, palatal collapse, and expiratory flow limitation have been added on page 15-16:

“Furthermore, Azarbarzin et al., were able to demonstrate in individuals with OSA, palatal prolapse into the velo/nasopharynx and causing expiratory flow limitation and a compensatory shunting of airflow through the mouth^27^. Taken all together, this could provide additional explanation as to why the mouth puffing phenomenon was found in the two studies by Jau et al., and that those with palatal collapse/prolapse would not benefit from mouth taping^1,12,27^. Therefore, for specific patient populations there is upstream or soft palate obstruction, mouth taping would appear to be an ineffective treatment. Additionally, Yang et al., have shown that for certain patients, namely those with high baseline mouth breathing during sleep and palatal obstruction, there are detrimental effects with forced mouth closure and that forced mouth closure during sleep is not universally beneficial^26^.”

## Critical Error on Page 16:

- The phrase **"limited upstream or soft palate obstruction"** suggests that mouth taping is beneficial for such patients, which **is incorrect.**
- The correct interpretation is that **mouth taping is ineffective in patients with significant upstream or soft palate obstruction.**
- **This is a critical error that must be corrected.**

This has been corrected on page 16:

“ Therefore, for specific patient populations there is upstream or soft palate obstruction, mouth taping would appear to be an ineffective treatment.”

## C5. Validity of Referencing Non-Empirical Discussion Points

- If the statement in **C5** is not based on direct research findings but rather reflects the authors' interpretation in the discussion, then **citing it as a reference must be reconsidered.**
- Discussion-based opinions from the original study authors should not be treated as empirical evidence.
- Please ensure that references used are **derived from actual study findings rather than speculative discussion points.**

Dear reviewer, you did not include which statement C5 is referring to, so we were not able to make a specific change.  As part of this revision we did carefully examine the manuscript and its references, and we are confident that the references cited in our paper are being used for review of existing study findings, as opposed to speculative discussion points.  If the reviewer has a specific statement in our paper that is of concern then please share the details and we are happy to address that instance, but in general we are comfortable that all references cited are consistent with accepted practice for systematic reviews.

**Conclusion**

The revised manuscript has improved, but several point should be addressed to ensure the accuracy and scientific integrity of the findings.

Key revisions needed:

- **Address study heterogeneity in the limitations section**
- **Correct the definition of mild OSA (AHI <15) on Page 13**
- **Revise the interpretation of mouth puffing and palatal prolapse on Page 16**
- **Correct the misleading statement that "limited upstream or soft palate obstruction" improves with mouth taping**
- **Re-evaluate the appropriateness of citing discussion-based opinions as references in C5**

Once these changes are made, the manuscript will be in a much stronger position.

Best regards,
